# Impact of Multi-View Fusion and Biomechanical Modeling on Markerless Motion Tracking

**DOI:** 10.1109/TBME.2025.3622032

**Published:** 2026-06

**Authors:** Zhixiong Li, Soyong Shin, Vu Phan, Evy Meinders, Eni Halilaj

**Affiliations:** Department of Mechanical Engineering, Carnegie Mellon University, USA; Department of Mechanical Engineering, Carnegie Mellon University, USA; Department of Mechanical Engineering, Carnegie Mellon University, USA; Department of Mechanical Engineering, Carnegie Mellon University, USA; Department of Mechanical Engineering, Carnegie Mellon University, Pittsburgh, PA 15213 USA

**Keywords:** Biomechanics, computer vision, kinematics, motion measurement

## Abstract

Markerless motion tracking using computer vision offers a scalable alternative to traditional marker-based analysis. While insight on how different emerging solutions compare could inform adoption across applications by highlighting accuracy-complexity tradeoffs, comprehensive benchmarking of these methods on the same open dataset remains a gap. This study evaluated thirteen single-view and two multi-view markerless motion capture methods against marker-based motion tracking for lower-extremity kinematics. Twenty-three healthy adults performed walking and lower-limb functional exercises, recorded with 20 infrared and 10 red-green-blue (RGB) cameras. The best-performing single-view model (WHAM) was used to test the hypothesis that the anatomical constraints imposed by biomechanical models improve the accuracy of vision-only solutions. We found that the addition of biomechanical modeling (bioWHAM) does not significantly improve overall kinematics accuracy, with root-mean-square differences (RMSD) sometimes being lower for the base-line model (WHAM) and sometimes for bioWHAM, with the median fluctuations across tasks being under 1.7°, in either direction. Multi-view methods generally outperformed single-view ones: OpenCap, an open-source solution using two cameras, outperformed WHAM by 1.7° (p < 0.0001), whereas Theia3D, a commercial software using ten cameras, outperformed OpenCap by 1.3° (p < 0.0001). These findings suggest that while multi-view systems can enhance accuracy, the marginal benefits may not justify the added complexity across all applications. In addition to helping users consider the hardware, software, and accuracy tradeoffs, these findings highlight the need for continued innovation in multi-view fusion and incorporation of biomechanical modeling and computer vision. The accompanying dataset (I-MOVE-23) should facilitate continued benchmarking of emerging solutions.

## I. Introduction

**Fig. 1.**
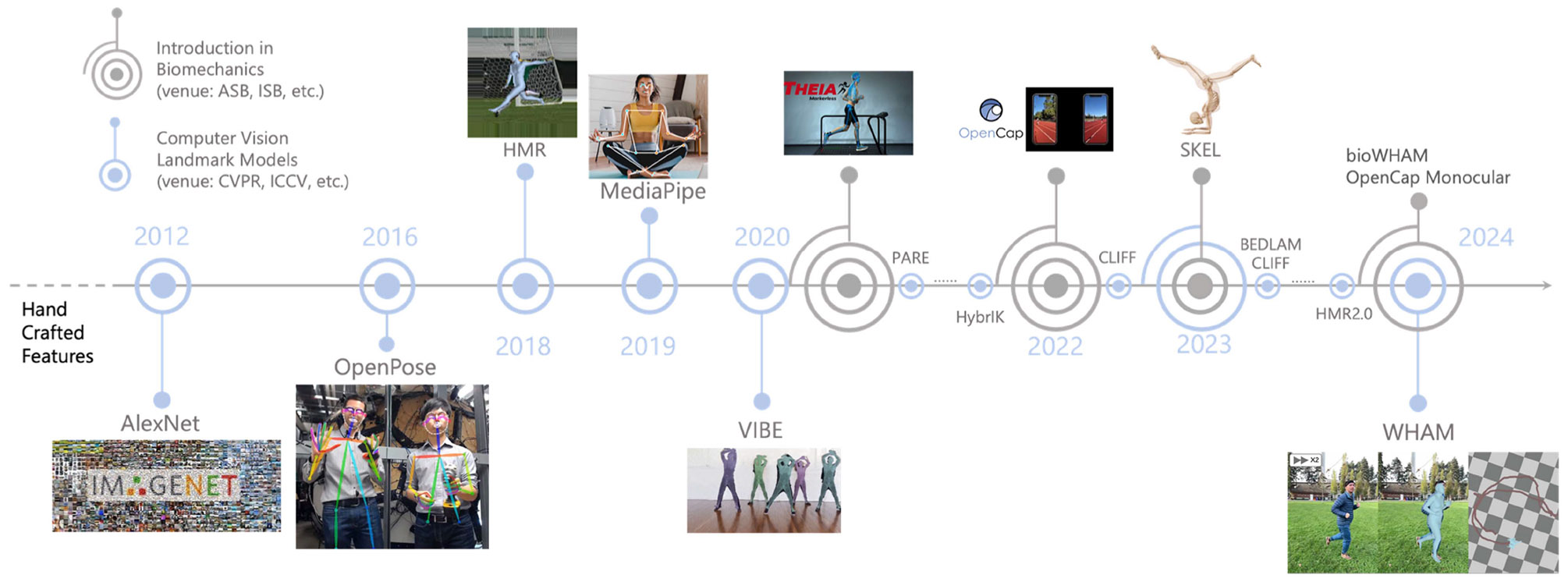
The decade of convergence: A timeline of computer vision meeting biomechanics. Computer vision breakthroughs from the last decade have laid the foundation for markerless motion tracking in biomechanics. From 2D joint center estimation to full 3D body pose and shape estimation, these methods are rapidly scaling access to quantitative assessments beyond the traditional biomechanics laboratory. While expertise was first flowing in one direction, with biomechanics solutions serving as wrappers around computer vision models, the last few years are marked by closer collaboration, wherein biomechanical models are being adopted in computer vision (e.g., SKEL [15], HSMR [16]). This growing interest at the intersection of the two fields should catalyze continued innovation that more directly addresses the needs of biomechanists.

VIDEO-BASED motion tracking, one type of markerless motion tracking, is modernizing and scaling motion analysis across applications, including sports analytics [1] and clinical gait analysis [2], [3]. Traditional marker-based motion capture has been challenging to scale due to equipment costs, the need for trained engineers, and markers that are not feasible to use in out-of-laboratory scenarios, such as studying athletes in the field. The deep learning boom of the early 2010s, catalyzed by the introduction of ImageNet [4] and propelled forward by the success of AlexNet [5], has transformed image- and video-based motion tracking, helping solutions move away from brittle approaches like hand-engineered features toward end-to-end learning. OpenPose [6], and similar approaches (Fig. 1), started a revolution that the field of biomechanics is embracing with cautious optimism and promising early demonstrations of clinical impact [2], [3].

While computer-vision approaches are evolving rapidly, from two-dimensional (2D) pose estimation [7] to model-based mesh recovery with 3D parametric body models [8], these advances have not been sufficiently synthesized for wider consumption within the field of human movement analysis, which is highly interdisciplinary. The accuracy of new computer-vision approaches is commonly tested using metrics of lesser relevance than kinematics to biomechanists, such as the Procrustes-Aligned Mean Per Joint Position Error (PA-MPJPE), making the value of new advancements in computer-vision approaches unclear. Unlike in other applications, such as human-robot interaction [9] or pedestrian detection [10], kinematic accuracy and physiological plausibility are critical in many biomechanics applications. However, despite being open, many computer vision tools require strong programming skills, making them largely inaccessible in biomechanics. Software with intuitive graphical user interfaces (GUIs), like Theia3D [11], [12], [13] and OpenCap [14], have made markerless motion tracking more accessible and user-friendly within biomechanics labs and clinical centers. However, users have to rely on these platforms to use the latest advances in computer vision and achieve improvements in accuracy. Theia3D is simple to use without computational skills, but users are not aware of how the software is evolving and whether it is taking advantage of developments in computer vision, due to its black-box nature. In addition, license costs and the need for at least six cameras further discourage wide adoption, especially in low-resource clinical and research environments. OpenCap is an open-source alternative. Expanding adoption, allowing more flexibility to use different camera setups, and offering opportunities for dynamic simulations is impactful. Despite these strengths, it is unclear if biomechanical models are improving kinematics-tracking accuracy over parametric body models [8] alone, which already include soft joint constraints. At a time when computer vision and biomechanics are coalescing more than ever before [15], [16], this is an important question to address.

Another question surrounding markerless motion tracking is how single-view solutions compare to multi-view ones. This is challenging to glean from the literature because advances in computer vision are focusing on single-camera (monocular) solutions, while biomechanics tools like OpenCap and Theia3D focus on multi-view ones. It is intuitive that multi-camera solutions should result in higher accuracy, but whether that improvement is currently sufficient to justify the time and inconvenience of calibrating and synchronizing multiple cameras, along with the data storage burden, is unclear. For example, a one-minute video captured at 100 Hz with one OptiTrack red-green-blue (RGB) camera generates approximately one gigabyte of data. Multiple activities captured by multiple cameras, at the large participant or patient numbers we aspire to capture, can lead to prohibitive data-storage requirements. In addition to helping users weigh the trade-offs, insights on current accuracy gains with multi-view approaches could motivate the development of more efficient cooperative fusion algorithms.

The goal of this work was to benchmark state-of-the-art approaches for markerless motion tracking, providing insight into the impact of biomechanical modeling and data fusion. Specifically, we hypothesized that sequential integration of a biomechanical model following deep learning solutions does not result in meaningful improvements in kinematics-estimation accuracy for activities of common interest in clinical biomechanics, such as walking and functional exercises. This hypothesis is based on the knowledge that many computer vision solutions already implement “soft” physiological constraints through pose priors that have learned the plausible ranges of motion through motion datasets [17], [18], such as AMASS [19]. These models include regularization terms that penalize poses that deviate from the learned pose distribution. Hard joint constraints, like those implemented in biomechanical models, may only be useful in extreme activities, such as yoga or acrobatics. We also hypothesized that current multi-view methods are more accurate than single-view ones, but not by large margins. This hypothesis is based on the knowledge that the computer vision literature is generally devoid of multi-view approaches. Last, we introduce a new open-access dataset for continued benchmarking of markerless motion tracking, titled I-MOVE-23 (**I**MU **and M**ulti-view **O**pen **V**ideo Data for **E**valuation of Markerless Tracking Methods in **23** Healthy Participants).

## II. Methods

### A. Data Collection

**Fig. 2.**
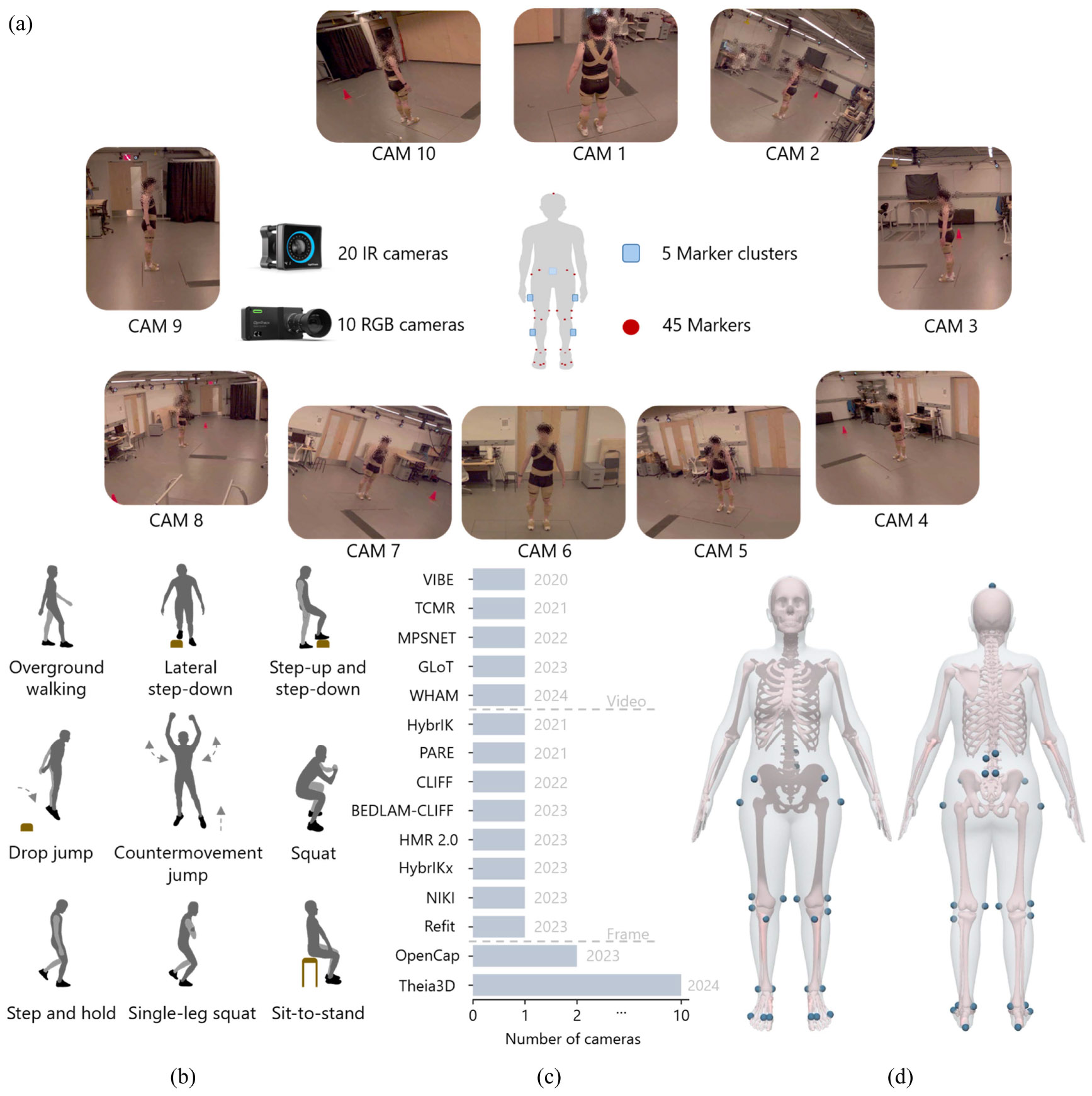
Overall study design. (a) Laboratory setup: A 1000-sqft lab equipped with both marker-based and vision-based systems, comprising 20 infrared (IR) and ten RGB cameras, was used to collect data from 23 healthy participants. (b) Activities monitored: Participants engaged in a variety of exercises, including a one-min session of overground walking, and three repetitions for lateral step-downs, step-ups and step-downs, drop jumps, countermovement jumps, squats, steps and holds, single-leg squats, and sit-to-stand movements for both legs. (c) Algorithms: Data analysis was performed using 13 single-camera methods (five video-based methods and eight frame-based methods) and two multi-camera methods utilizing configurations of two cameras (OpenCap) and ten cameras (Theia3D). (d) Virtual marker placements: A total of 29 virtual markers were manually attached on the SMPL output for OpenSim inverse kinematics estimation. The number of markers is different from that of marker-based motion capture experiments because meshed models do not require tracking markers.

Twenty-three healthy participants (10 females, 13 males; mean age = 36 ± 11 years, height = 1.73 ± 0.09 m, weight = 77.2 ± 18.6 kg) were recruited after obtaining approval from the Institutional Review Board (IRB) of Carnegie Mellon University and informed consent. Motion data were collected using both marker-based and markerless motion capture systems (Fig. 2(a)). All recordings were performed in a controlled indoor environment with consistent ambient lighting across sessions. Ten RGB cameras (OptiTrack, Corvallis, OR) were used to record video data at 100 Hz for markerless motion analysis. A total of 20 infrared (IR) cameras (OptiTrack, Corvallis, OR) were used to track motion at 100 Hz with a modified Rizzoli markerset consisting of 45 retroreflective markers, which served as the reference for comparison. The cameras were synchronized temporally using OptiTrack’s software. Before each data collection session, IR cameras were calibrated spatially using a calibration wand (OptiTrack, Corvallis, OR), whereas RGB cameras were calibrated using a calibration board (Theia Markerless Inc., Kingston, ON, Canada). Spatial calibration accuracy was verified by ensuring that the root-mean-square error of the calibration board corners remained below 2 mm.

Participants were then recorded while performing at least one minute of overground walking at a self-selected speed and eight lower-extremity exercises, including lateral step-down, step-up and step-down, drop jump, countermovement jump, squat, step and hold, single-leg squat, and sit-to-stand, each for a minimum of three successful repetitions (Fig. 2(b) ). Data from the same experimental protocol were used across all the markerless motion tracking methods.

### B. Data Processing

We obtained ground-truth kinematics using OpenSim’s Inverse Kinematics (IK) solver, with the Rajagopal model [20]. Thirteen single-view and two multi-view computer-vision algorithms were selected for comparison (Fig. 2(c) ). The single-view algorithms were based on their GitHub popularity, academic citations, and performance on benchmark computer-vision datasets. These methods included both frame-based [21], [22], [23], [24], [25], [26], [27], [28] and video-based [29], [30], [31], [32], [17] approaches. To ensure consistency with the frame rates of the videos on which the video-based tools were trained, we downsampled the data to 33 Hz before applying video-based methods. Kinematics were evaluated and expressed in the context of a parametric meshed model of the human body, the Skinned Multi-Person Linear (SMPL) model [8]. This statistical model is parametrized in terms of 10 body-shape and 72 body-pose parameters, with joint coordinate systems roughly following anatomical axes, but deviating from the Grood and Suntay convention [33], [34]. We also selected two multi-view methods, Theia3D (Theia Markerless Inc., Kingston, ON, Canada) and OpenCap [14], based on their popularity within the movement sciences literature. For Theia3D, which requires a minimum of six cameras, we utilized all ten cameras. For OpenCap, which requires a minimum of two cameras, we used two cameras, positioned at approximately 45° relative to the subject whenever possible [14]. This decision was made after a preliminary analysis confirming no accuracy gains beyond two cameras. Both multi-view methods used the same calibration files, ensuring consistent extrinsic and intrinsic parameter estimation across all recordings.

For single-view methods, we evaluated each method across all ten cameras and used average root-mean-square differences (RMSD) across views for comparison across methods. However, when comparing single- and multi-view methods for hypotheses 1 and 2, we selected the best-performing camera—Camera 7—which was positioned at 45°, on the front right side of the subjects across all the exercises (Fig. 2(a)). Although we selected the same camera for walking, the position of the camera relative to the subject was changing given the nature of overground walking. We excluded overground walking trials for two subjects because their initial starting positions and orientations were near the edge of one of the two cameras used to obtain OpenCap kinematics.

To explore the effect of applying a biomechanical model after the computer-vision solution, we placed virtual markers on the SMPL mesh, simulating the placement of physical markers used in traditional motion capture. This process enabled mapping of the estimated body motion onto a biomechanical model, facilitating evaluation of biomechanically constrained inverse kinematics. To test our hypothesis, we selected WHAM, the best-performing single-view model and hereon refer to its enhanced biomechanical output as bioWHAM (Fig. 2(d)). The same experienced biomechanist who placed the markers during data collection also manually identified marker locations on the meshed body model, following anatomical landmarks. In total, 29 virtual markers were selected and used to perform inverse kinematics in OpenSim [35]. We used a sparser markerset than the one in the marker-based analysis because tracking-cluster markers are not necessary in the vision-based analysis.

Both marker data and kinematics output from markerless methods were filtered using a 4th-order low-pass Butterworth filter with a cutoff frequency of 6 Hz as a zero-lag filter. To remove any potential bias arising from mismatches in anatomical coordinate systems between the marker-based and markerless methods, we used the middle two seconds of the static pose before each exercise to determine the static pose transformations (e.g., SMPL-to-OpenSim or Theia3D-to-OpenSim or OpenCap-to-OpenSim). These transformations were used to align the anatomical coordinate system in subsequent activities to ensure consistent alignment for each method.

### C. Statistical Analyses

We tested hypotheses 1 and 2 simultaneously using a Linear Mixed-Effects Model, treating motion-capture methods, joints (ankle, knee, hip), and activities as fixed effects and sex, age, BMI, weight, and height as covariates. A random intercept for the subject was included to account for repeated measures within individuals. Because the RMSD distribution violated normality assumptions (Shapiro-Wilk test, p < 0.0001), a Box-Cox transformation was applied, which identified a log transformation (*λ ≈* 0) as appropriate to better meet the residual normality assumption. Accordingly, all the analyses were conducted using the log-transformed RMSD as the outcome. To evaluate main effects and interactions, we used Type III ANOVA with Satterthwaite’s approximation. Post-hoc pairwise comparisons between methods were conducted using estimated marginal means. A significance threshold of p = 0.05 was applied for hypothesis 1, while Bonferroni correction was applied to adjust for three comparisons (p = 0.0167) for hypothesis 2. Last, correlation plots were generated to explore associations between biological variables and RMSD, with the significance level corrected for multiple comparisons (p = 0.01). Results are reported as medians with first and third quartiles. Analyses were conducted with the use of the R software packages lme4, lmerTest, and emmeans [36], [37], [38], [39].

In addition to addressing the two primary hypotheses, we also answered auxiliary questions. First, to select the best single-view method(s) to be used for comparison with multi-view methods, we used the Kruskal-Wallis test, followed by post-hoc Wilcoxon signed-rank tests. We ranked the single-view methods in order of increasing RMSD and performed pair-wise comparisons across neighbors until a significant increase in RMSD was observed, at which point we stopped, declaring all prior methods as better performers. In a second auxiliary analysis, we set out to determine whether outcomes of relevance in a biomechanics context (i.e., joint kinematics) are well-correlated with outcomes commonly used in the computer-vision literature, such as PA-MPJPE. The relationship between kinematics RMSD and PA-MPJPE was assessed using Spearman correlation coefficient, using a standard computer vision benchmark dataset, 3DPW [40], and the data collected as part of this study.

## III. Results

### A. Biomechanical Modeling

**Fig. 3.**
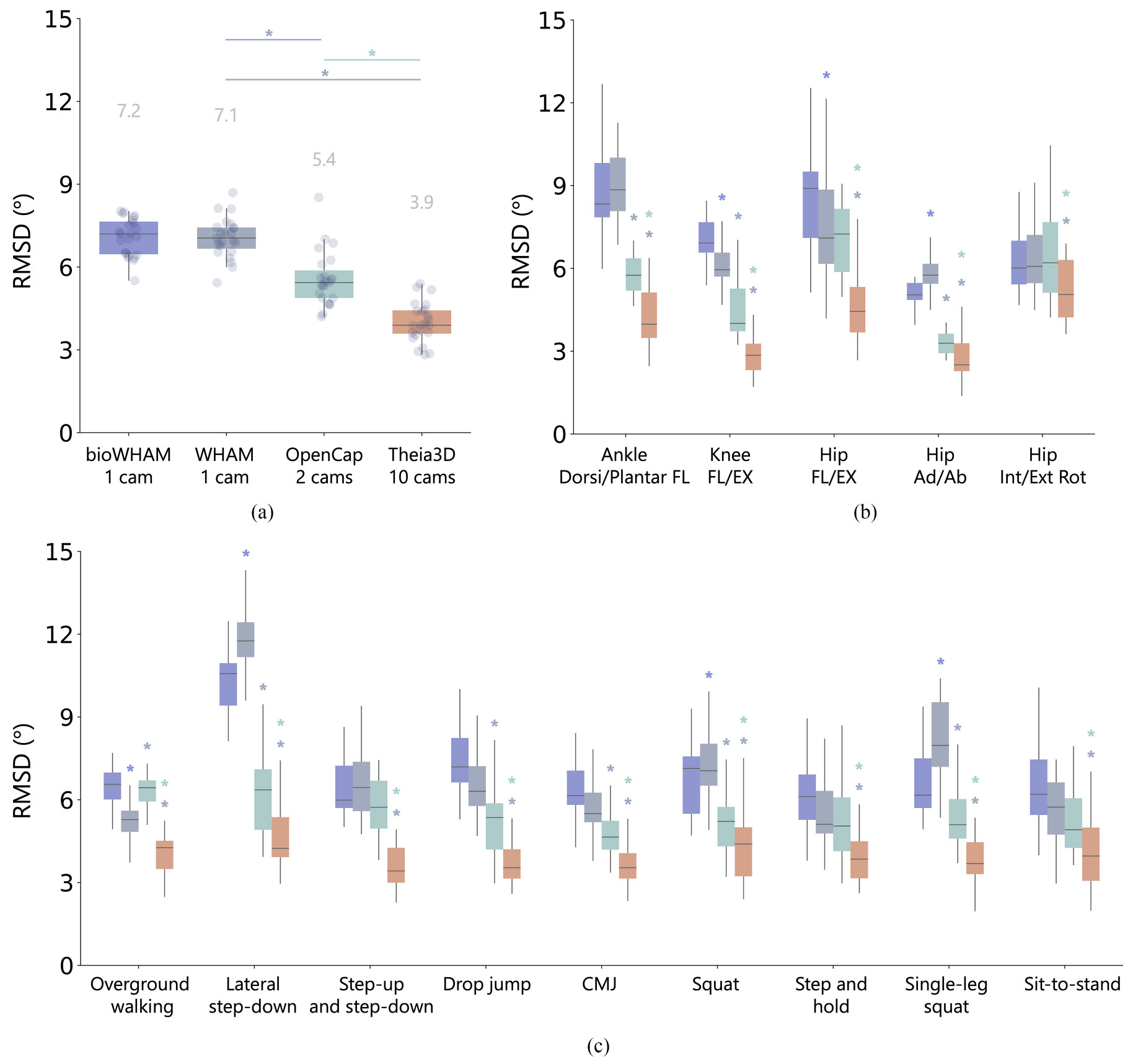
Performance by method, degree of freedom, and activity. A comparison of bioWHAM and WHAM helped test the first hypothesis, revealing that (a) overall, sequential implementation of a biomechanical model does not improve kinematics accuracy over computer-vision algorithms alone. The benefits of a biomechanical model were not consistent across (b) degrees of freedom and (c) activities, with some showing WHAM and some bioWHAM to be superior. Generally, multi-view approaches like OpenCap and Theia3D were more accurate than single-view ones, confirming the second hypothesis. (*: p < 0.05 for hypothesis 1 and p < 0.0167 for hypothesis 2; CMJ: Countermovement Jump, FL: Flexion, EX: Extension, Ad/Ab: Adduction/Abduction, Int/Ext Rot: Internal/External Rotation).

Sequential implementation of a biomechanical model did not systematically improve motion-tracking accuracy across degrees of freedom and activities, but it did in specific cases. Overall, WHAM and bioWHAM demonstrated comparable performance (0.0° [−0.5°, 0.4°]; p = 0.3166) (Fig. 3(a)). When analyzed by joint, bioWHAM and WHAM performed similarly in ankle dorsi/plantar flexion (−0.8° [−1.5°, 0.8°]; p = 0.0884) and hip internal/external rotation (−0.2° [−0.5°, 0.2°]; p = 0.6287). bioWHAM demonstrated higher RMSDs in knee flexion/extension by 0.6° ([0.4°, 1.4°]; p = 0.0001) and hip flexion/extension by 0.8° ([−0.7°, 2.2°]; p = 0.0013), and lower RMSDs in hip adduction/abduction by 0.6° ([−1.1°, −0.2°]; p = 0.0082) (Fig. 3(b)). When analyzed by activity, bioWHAM demonstrated lower RMSDs than WHAM during single-leg squat by 1.6° ([−1.8°, −1.2°]; p < 0.0001), lateral step-down by 1.7° ([−2.0°, −1.2°]; p = 0.0122), and squat by 0.5° ([−1.4°, 0.3°]; p = 0.0259) (Fig. 3(c)). Conversely, bioWHAM showed higher RMSDs in walking by 1.2° ([1.1°, 1.5°]; p < 0.0001) (Fig. 3(c)). In all the other activities, including step-up and step-down (p = 0.9211), drop jump (p = 0.0577), countermovement jump (p = 0.1206), step and hold (p = 0.1113), and sit-to-stand (p = 0.0652), no significant differences were observed between bioWHAM and WHAM (Fig. 3(c)). Marker scaling errors were comparable between marker-based and markerless analyses (p = 0.1341), whereas tracking errors were slightly higher for bioWHAM than marker-based analyses with a median difference of 0.23 cm ([−0.10 cm, 0.44 cm]; p = 0.0021).

### B. Single-View Versus Multi-View

**Fig. 4.**
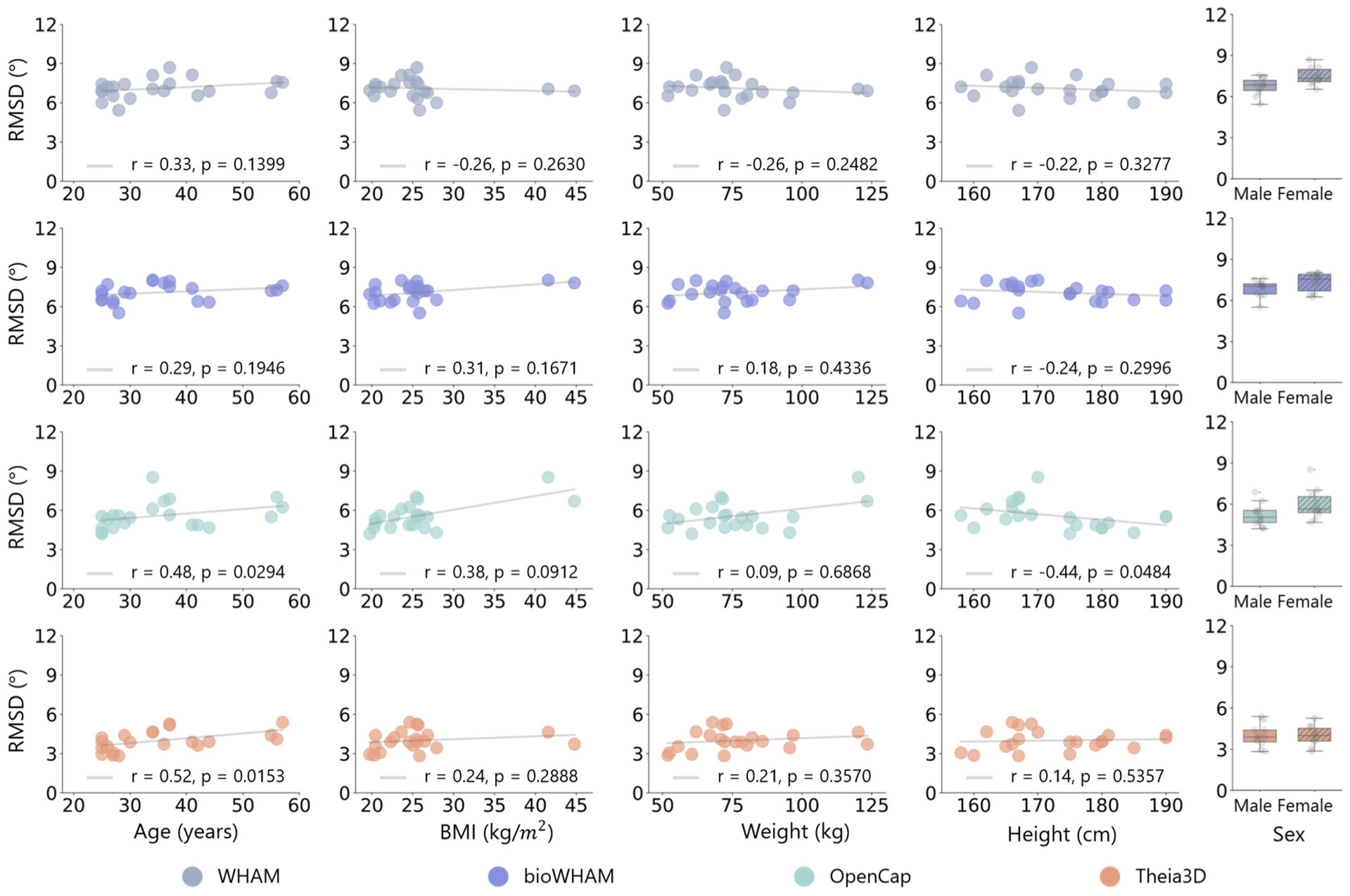
Effects of biological variables. After performing Bonferroni correction for the 5 tests on the effect of age, BMI, weight, height, and sex (p = 0.01), none of the biological variables had a significant effect on tracking accuracy, across the four methods.

Multi-view methods outperformed single-view ones, across degrees of freedom and activities (see Supplementary Fig. S1 for time-normalized joint angle trajectories across all methods). Theia3D achieved lower RMSDs than OpenCap by a median difference of 1.3° [0.9°, 1.8°] and WHAM by 3.0° [2.6°, 3.5°] (p < 0.0001) (Fig. 3(a)). OpenCap demonstrated lower RMSDs than WHAM in ankle dorsi/plantar flexion by 3.1° [−4.2°, −2.3°], knee flexion/extension by 1.7° [−2.1°, −1.3°], and hip adduction/abduction by 2.8° [−3.0°, −2.1°] (p < 0.0001), but not in hip internal/external rotation (p = 0.6969) and hip flexion/extension (p = 0.9911) (Fig. 3(b)). Across activities, Theia3D achieved lower RMSDs than WHAM by 1.3° to 7.5° and OpenCap by 0.5° to 2.4° (p = 0.0004 vs. OpenCap during squat; p < 0.0001 for all others) (Fig. 3(c)). OpenCap showed lower RMSDs than WHAM in lateral step-down by 5.5° ([−6.9°, −5.4°]; p < 0.0001), drop jump by 1.4° ([−1.9°, −0.4°]; p = 0.0001), countermovement jump by 1.3° ([−1.9°, −0.3°]; p < 0.0001), squat by 2.3° ([−3.2°, −1.0°]; p < 0.0001), and single-leg squat by 2.8° ([−3.8°, −1.6°]; p < 0.0001). No significant differences were found in step-up and step-down (p = 0.0388), step and hold (p = 0.0193), and sit-to-stand (p = 0.0171). Notably, WHAM outperformed OpenCap in walking by 1.2° ([−1.7°, −0.5°]; p < 0.0001). Overall, biological variables showed no significant association with RMSD across methods (Fig. 4). Two high-BMI individuals were retained in these analyses because despite being outliers in terms of BMI, they were not outliers in terms of kinematic RMSD. They were also not influential points.

### C. Auxiliary Findings

**Fig. 5.**
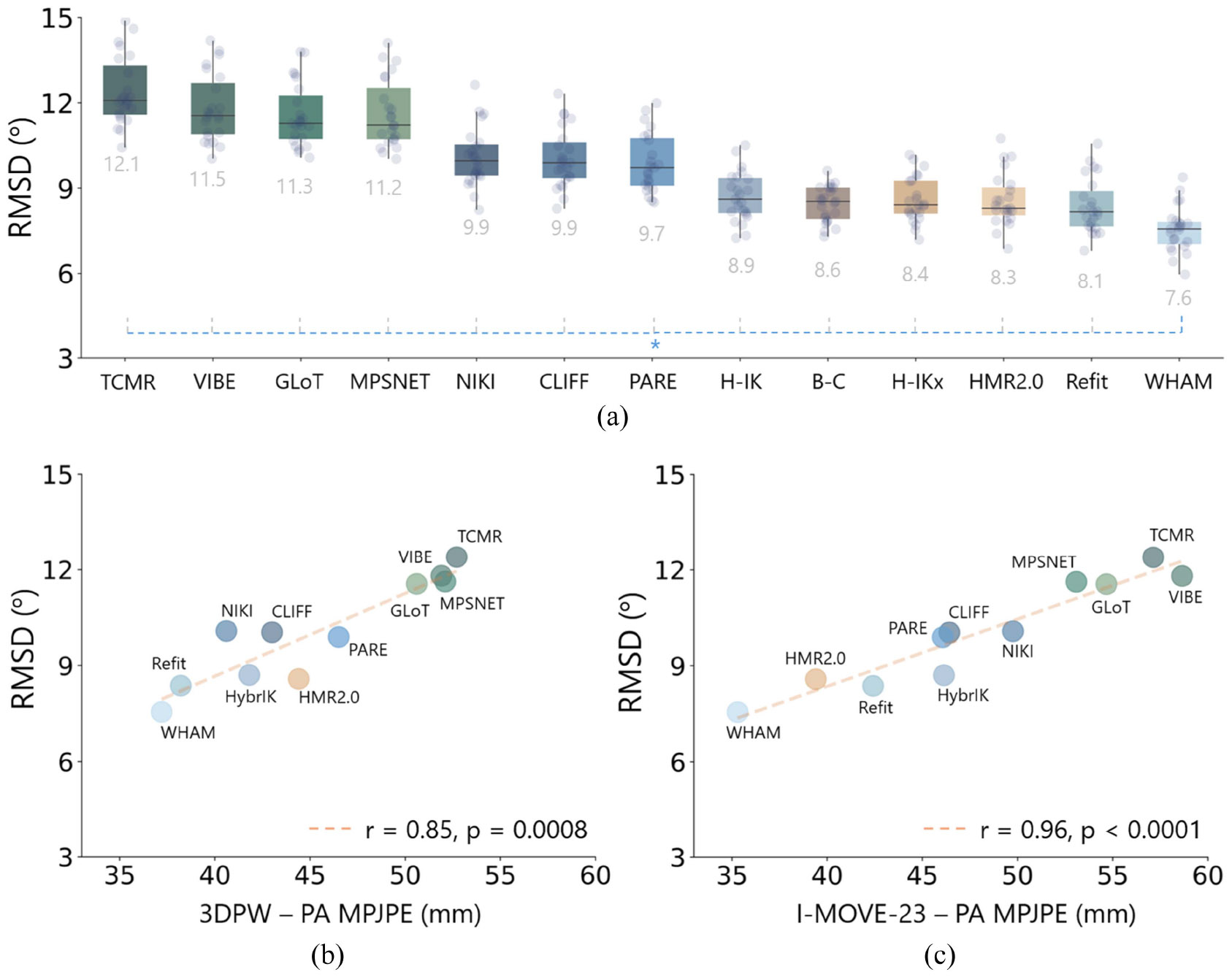
Single-view method performance. (a) WHAM achieved the lowest RMSD across the tested single-view methods. Results here represent means across all ten camera views. (b) A strong correlation was observed between kinematics and Procrustes Aligned Mean Per Joint Position Error (PA-MPJPE), a metric that is commonly used to benchmark computer-vision methods. Here, both metrics were computed on the 3DPW dataset, a standard benchmark dataset in computer vision. (c) A similarly strong correlation was observed when using the data recorded as part of this study. (H-IK: HybrIK, B-C: BEDLAM CLIFF, H-IKx: HybrIKx).

Among single-view methods, WHAM achieved the lowest RMSD, outperforming the next most accurate method by 0.8° ([−1.0°, −0.7°]; p < 0.0001) and the least accurate method by 4.8° ([−5.3°, −4.5°]; p < 0.0001) (Fig. 5(a)). Kinematics accuracy across methods was highly correlated with PA-MPJPE, when using both the 3DPW benchmark dataset (r = 0.85; p = 0.0008; Fig. 5(b)) and the dataset collected as part of this study (r = 0.96; p < 0.0001) (Fig. 5(c)).

## IV. Discussion

The goal of this study was to provide insight into the hardware, software, and accuracy tradeoffs of different markerless motion tracking solutions. We found that the application of a biomechanical model sequentially after computer-vision models does not systematically improve joint-kinematics estimation accuracy. We also found that current multi-view methods are generally more accurate than single-view ones, by 1.7° when using two cameras (OpenCap) and by 3.0° when using ten cameras (Theia3D). Multi-view methods also exhibited narrower limits of agreement, less scatter, and reduced heteroscedasticity than single-view ones (see Supplementary Fig. S2-6), which has implications for data analysis and interpretation. The burden of additional cameras, setup time for spatial calibration, and data-storage requirements are not negligible, however, and should be considered against these accuracy gains. The commercial solution, Theia3D using ten cameras, outperformed the open-source one, OpenCap using two cameras, by 1.3°, helping place in context the license cost versus accuracy gains.

When interpreting the findings reported here, the following limitations should be considered. We only benchmarked the aforementioned methods on participants without gait impairments. While future work should continue testing these methods in patient populations, as RMSDs may slightly vary in other populations and settings, we have no reason to believe that the conclusions drawn here about the impact of biomechanical modeling and multi-view approaches would not be valid more broadly. We also tested these methods in a laboratory setting, without researchers or clinicians in the field of view, as it may be the case in clinical settings. It is therefore important to note that these findings are only relevant to single-person data capture and that applications with more complex backgrounds or where multiple persons are in the field of view require separate benchmarking. Last, we tested these methods during walking and a set of functional exercises used in lower-extremity rehabilitation. While those interested in capturing other activities should perform independent validation, we believe that the conclusions on biomechanical modeling and data fusion will generally hold true, except for activities with extreme ranges of motion.

Contrary to prevailing assumptions in both the computer-vision and biomechanics communities, we found that applying a biomechanical model does not systematically improve kinematics accuracy. This result challenges the intuition that biomechanical modeling, involving inverse kinematics with hard joint constraints, should refine the outputs of vision-based solutions by enforcing anatomical feasibility. One possible explanation is that methods that use parametric mesh models like SMPL [8], already impose strong priors on body shape and pose via learned statistical distributions and differentiable kinematic chains, which act as soft constraints during optimization. These priors, trained on relatively large and diverse data [19], may effectively regularize joint motion in a way that leaves limited room for improvement through subsequent biomechanical modeling (see Supplementary Fig. S7 for pairwise comparison across individual camera views). It is important to note that our hypothesis was only tested on mesh-based approaches and that this finding may not generalize to tools like OpenCap and Theia3D, which do not fit meshed models to video data. Prior work [22] has shown that mesh-based approaches, while offering more information, also result in slightly less accurate joint-center estimates. Additionally, biomechanical models offer the benefit of enabling neuromus-culoskeletal simulations for estimation of quantities that cannot be measured directly, such as moments and forces. A more nuanced, application-specific integration strategy may therefore be appropriate. We support the expansion of similar ablation studies that shed light on the contributions of each layer of computation. Efforts to add biomechanical models to mesh-based approaches are gaining traction [16], and these ablation experiments will help isolate the specific benefits of each proposed contribution.

Despite the intuitive assumption that multi-view systems should be more accurate, and our findings confirming this intuition, gains of 2 to 3 degrees over single-view methods may not always justify the inconvenience of multi-view systems. This limited gain could be partly explained by a disconnect between research priorities in computer vision and biomechanics. The computer-vision community has largely focused on monocular pose estimation, driven by the availability of large single-view datasets and the goal of maximizing applicability in uncontrolled environments. As a result, algorithmic innovation in multi-view fusion has lagged behind, with many multi-view pipelines relying on simple triangulation rather than advanced cooperationfusion strategies. Meanwhile, the biomechanics community, though more invested in multi-view systems, has often adopted existing computer-vision models without developing new fusion techniques tailored to our accuracy needs. This disconnect has left a gap where the full potential of multi-view systems remains unrealized. Addressing this gap will require continued innovation in multi-view data fusion.

The auxiliary finding reported here that joint-kinematics accuracy and metrics used within the computer-vision community are highly correlated will help with evaluation of new methods in this rapidly developing field. Biomechanics studies take years, while new computer-vision methods are published yearly at a rapid pace that makes it difficult for biomechanists to quickly assess if a new method provides sufficient improvement to warrant adoption over an existing method. By publishing this regression model, as well as all the data and code, we hope to make this assessment more practical. Nevertheless, adoption of joint kinematics accuracy should also be a target for the computer-vision community as the two fields continue to overlap.

## V. Conclusion

The impact of markerless motion tracking has already been illustrated in clinical and sports applications [2], [3], even with approaches less accurate than those available today. Not all applications require high accuracy. Not all scientific questions can be answered using a single smartphone. The goal of this work, and future work of similar nature, is to provide systematic insight into how the growing number of markerless motion tracking tools, both open source and commercial, compare, so that researchers can make informed decisions. Another goal is to provide an open dataset on which future methods can be tested. We also provide open code so that the same methods can be tested in other populations and laboratories, available at the I-MOVE-23 website: https://sites.google.com/andrew.cmu.edu/i-move-23/home.

## Supplementary Material

supp1-3622032

This article has supplementary downloadable material available at https://doi.org/10.1109/TBME.2025.3622032, provided by the authors.
