# Supplementary material for: Impact of Multi-View Fusion and Biomechanical Modeling on Markerless Motion Tracking
Source: IEEE Trans Biomed Eng. Author manuscript; Available in PMC 2026 Jul 22. (PMC13389856; doi:10.1109/TBME.2025.3622032)
Supplement: supp1-3622032 [file NIHMS2179440-supplement-supp1-3622032.docx]

Supplementary Material (IEEE – TBME)


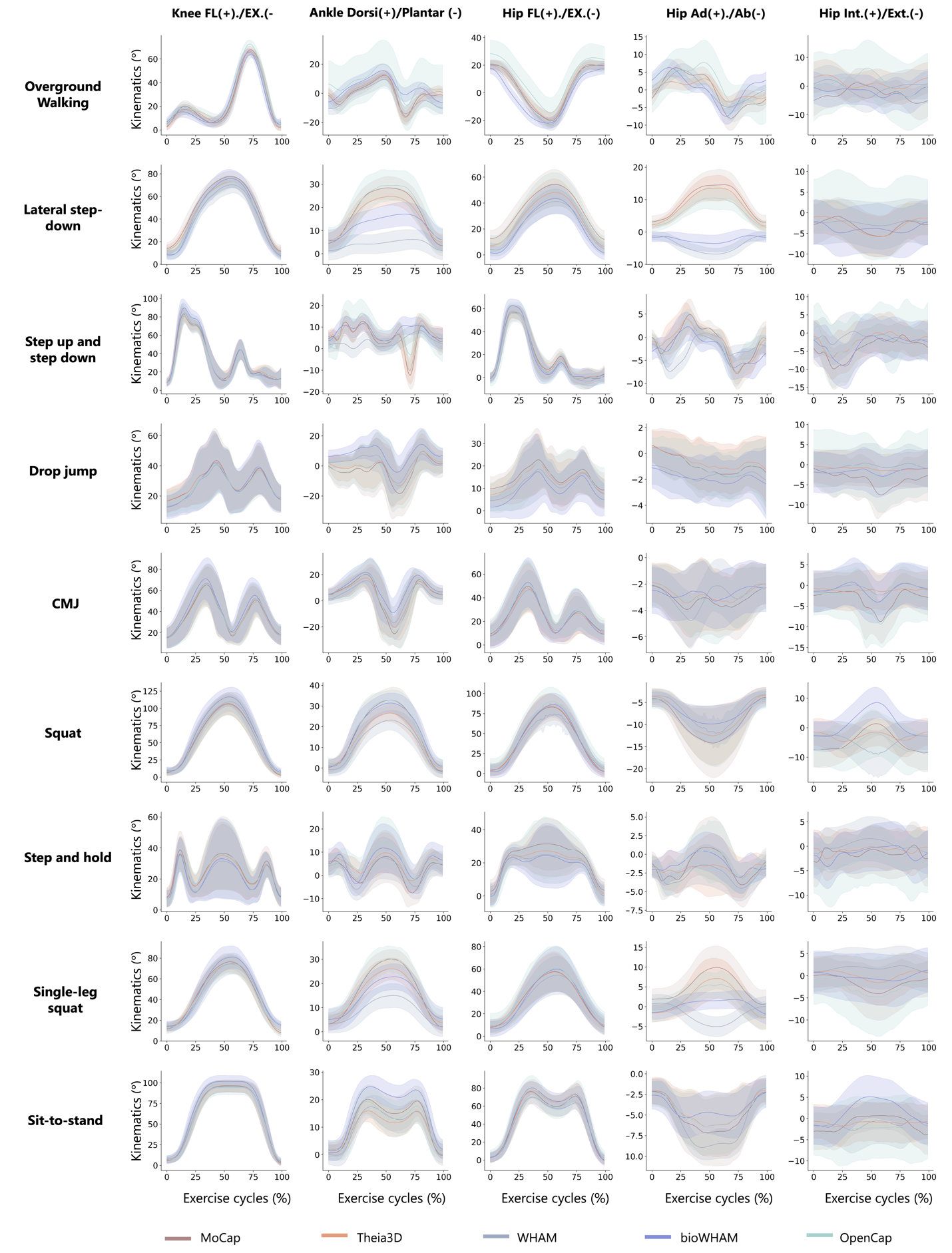


Fig. S1.  Kinematic Trajectories Across Methods. Multi-camera methods generally outperformed single-camera methods across different exercises and joint kinematics, particularly for ankle kinematics. However, single-view methods demonstrated high accuracy in hip and knee flexion. Secondary degrees of freedom in the hip often exhibited higher RMSD.


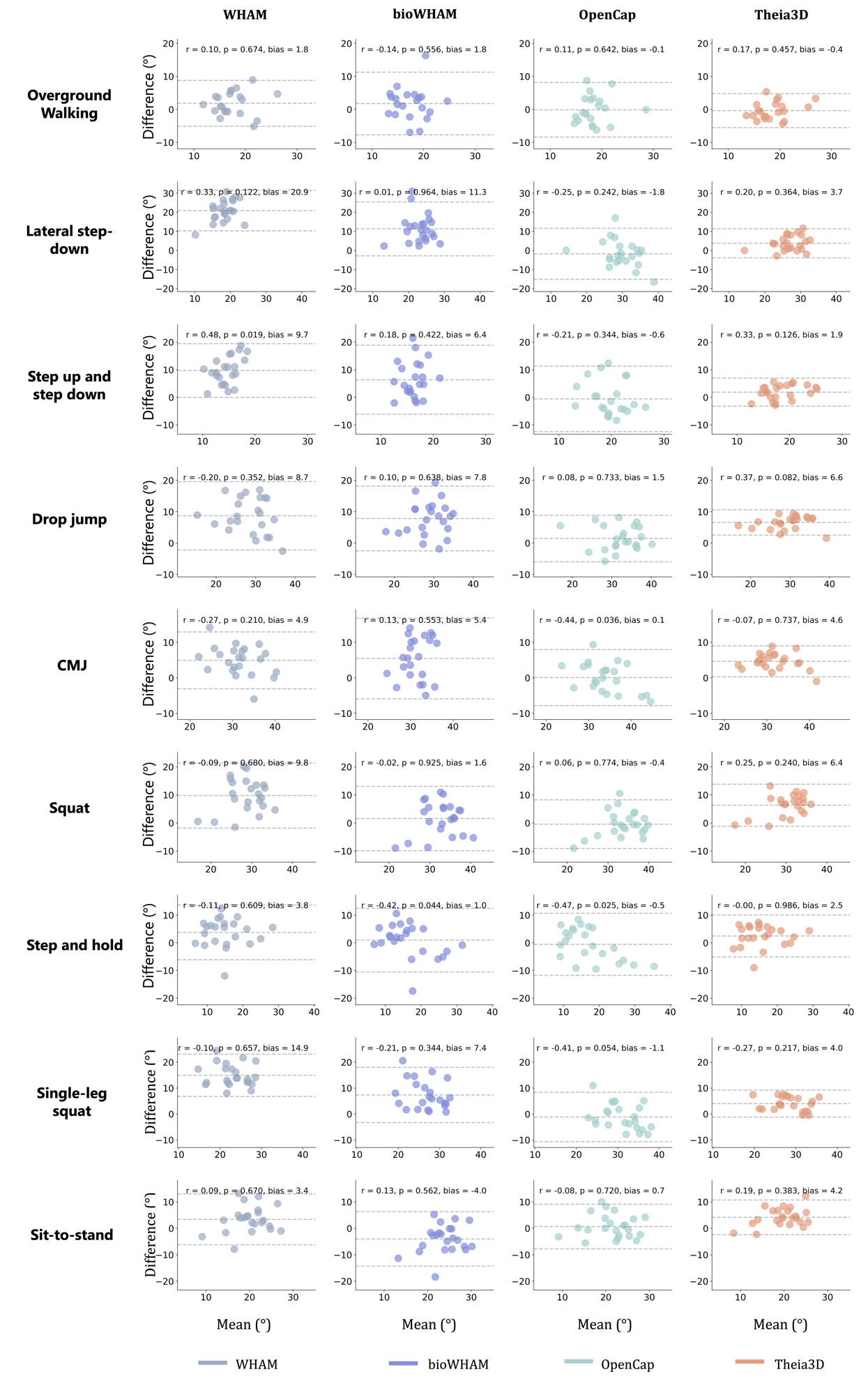


Fig. S2.  Markerless and Marker-Based Agreement: Ankle Dorsi/Plantar flexion. WHAM showed the largest range of bias and the widest limits of agreement, indicating lower agreement and greater random error in peak joint angle estimates. bioWHAM did not consistently improve this bias or tighten the limits of agreement. Theia3D showed higher bias than OpenCap, but both demonstrated generally narrower limits of agreement. Overall, agreement and limits of agreement varied more by activity than by method, with no consistent link between bias and peak values.


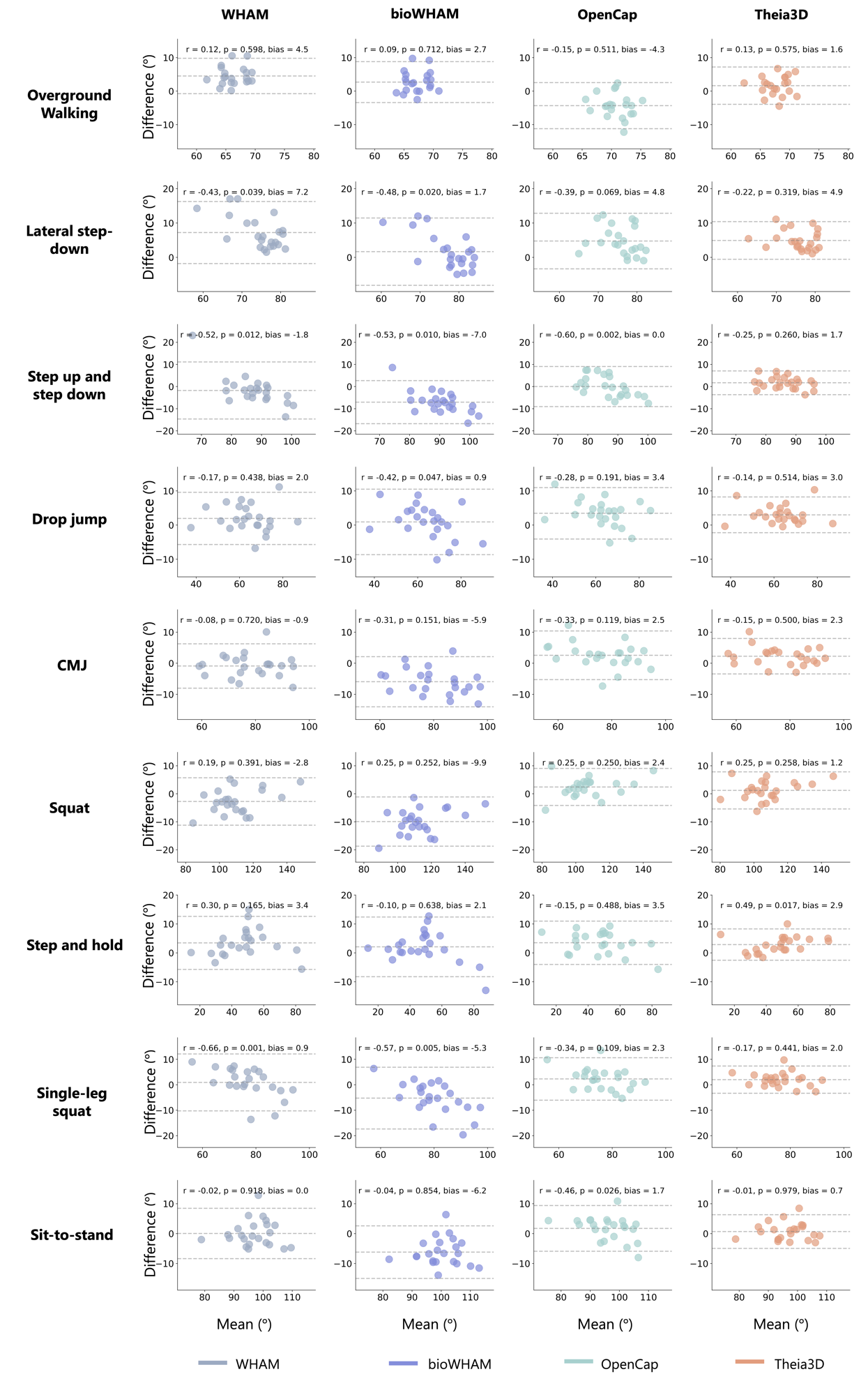


Fig. S3.  Markerless and Marker-Based Agreement: Knee Flexion/Extension. The peak knee flexion/extension angle for WHAM generally showed comparable bias to OpenCap and Theia3D, except in lateral step-down, remaining within a $\pm$5$^{\circ}$ range. However, the limits of agreement were generally wider for single-view methods, with the exception of overground walking, compared to multi-view methods. bioWHAM introduced greater bias and did not consistently reduce the limits of agreement. Heteroscedasticity was present in several tasks for single-view approaches. These findings highlight that, while multi-view systems often reduce bias and improve agreement consistency, single-view methods may still achieve comparable performance in tasks such as overground walking.


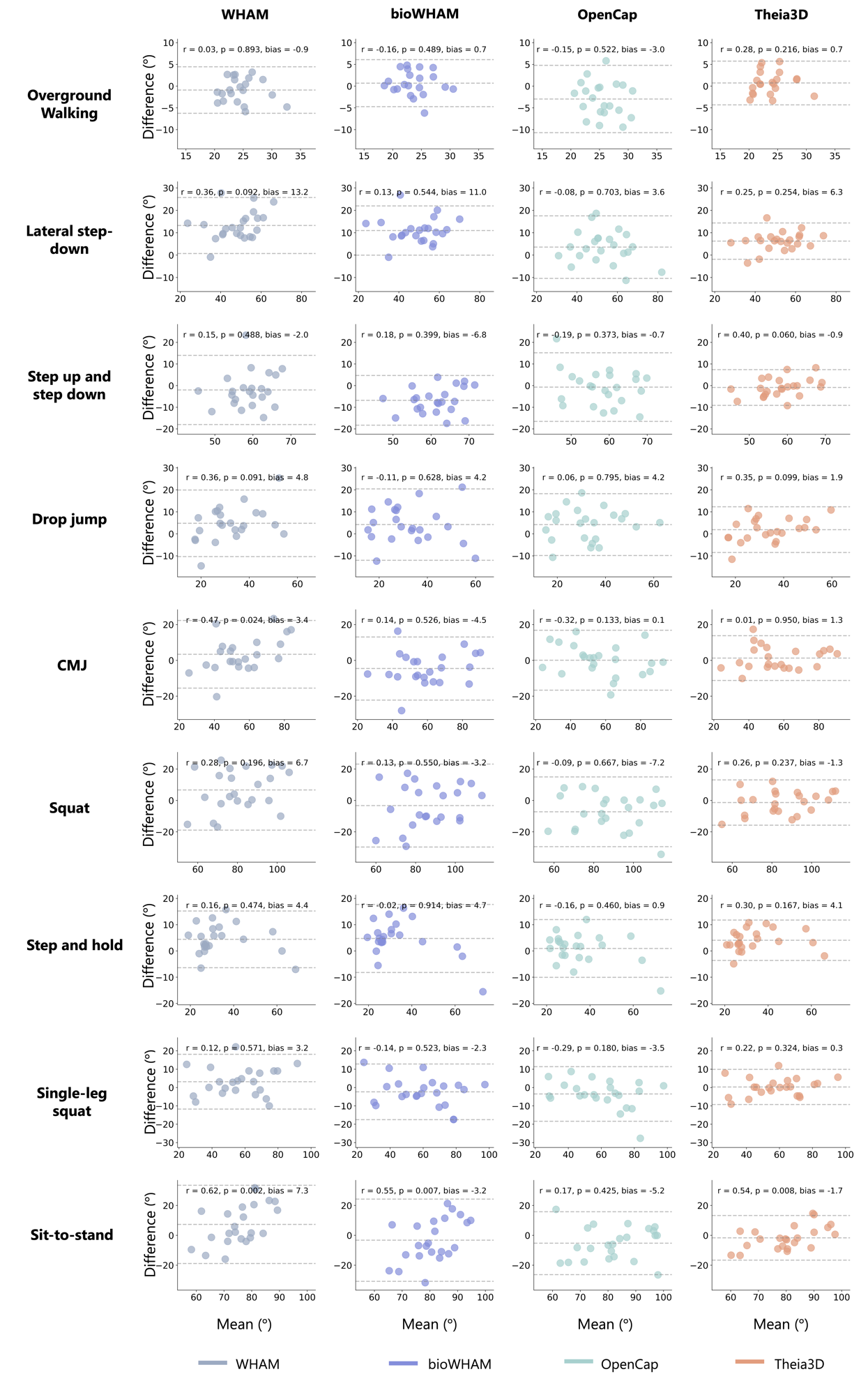


Fig. S4.  Markerless and Marker-Based Agreement: Hip Flexion/Extension. The peak hip flexion/extension angles for WHAM, OpenCap, and Theia3D showed different levels of bias across tasks, generally within $\pm$5$^{\circ},$ range, except during lateral step-down and squat, where it was greater. bioWHAM often introduced additional bias relative to WHAM and did not consistently reduce the limits of agreement. Random error for single-view methods was comparable to those of OpenCap, but remained wider than that of Theia3D.


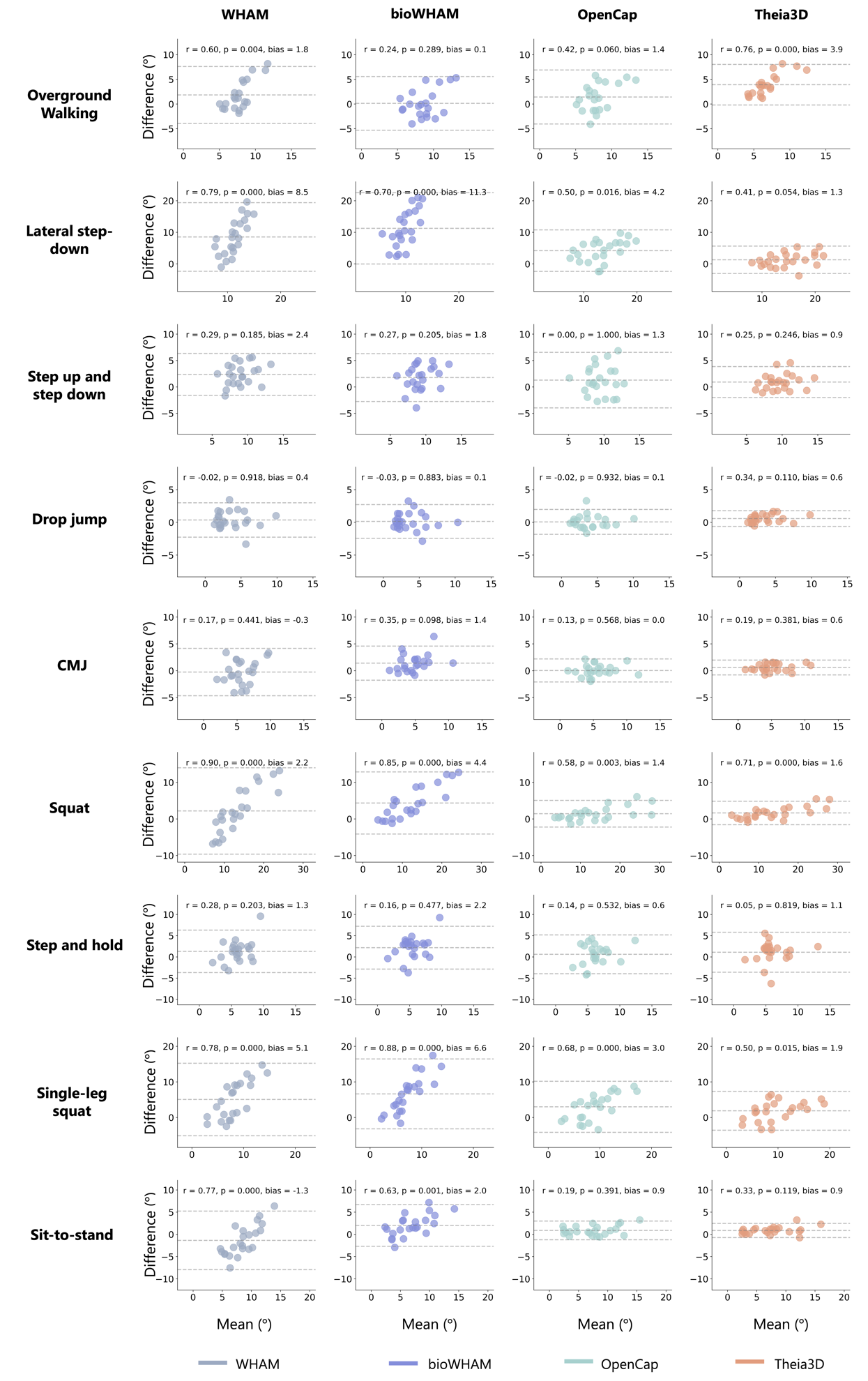


Fig. S5.  Markerless and Marker-Based Agreement: Hip Adduction/Abduction. The peak hip adduction/abduction angle bias was generally higher in single-view methods than multi-view methods. Bias for multi-view methods remained mostly within a $\pm$5$^{\circ}$ range, and both multi-view methods demonstrated narrower limits of agreement and thus lower random variability than single-view methods. Heteroscedasticity was present in squat-related exercises across nearly all methods, in sit-to-stand tasks for single-view methods, and in overground walking for WHAM and Theia3D, suggesting increased bias at higher peak values.


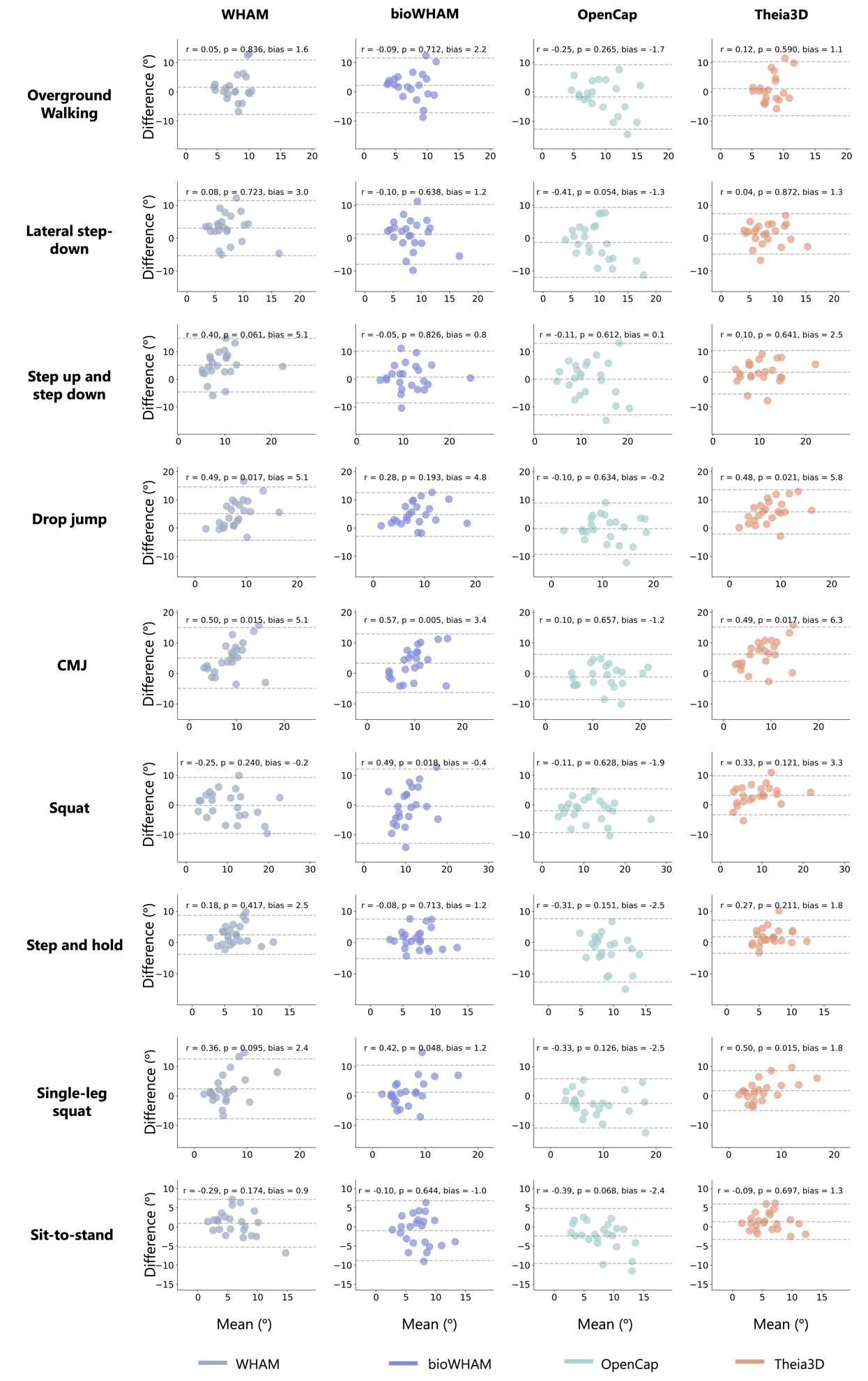


Fig. S6.  Markerless and Marker-Based Agreement: Hip Internal/External Rotation. The peak hip internal/external rotation angles for WHAM and bioWHAM showed variable bias across tasks, without a consistent trend. OpenCap generally led to underestimation whereas Theia3D tended to overestimate. Bias was mostly within a $\pm$5$^{\circ}$ range. However, OpenCap did not significantly reduce the limits of agreement compared to single-view methods, and in some cases, exhibited even greater variability. Theia3D demonstrated narrower limits of agreement in some tasks but not consistently across activities. Heteroscedasticity was present in some dynamic tasks for Theia3D and WHAM, such as the countermovement jump and drop jump.


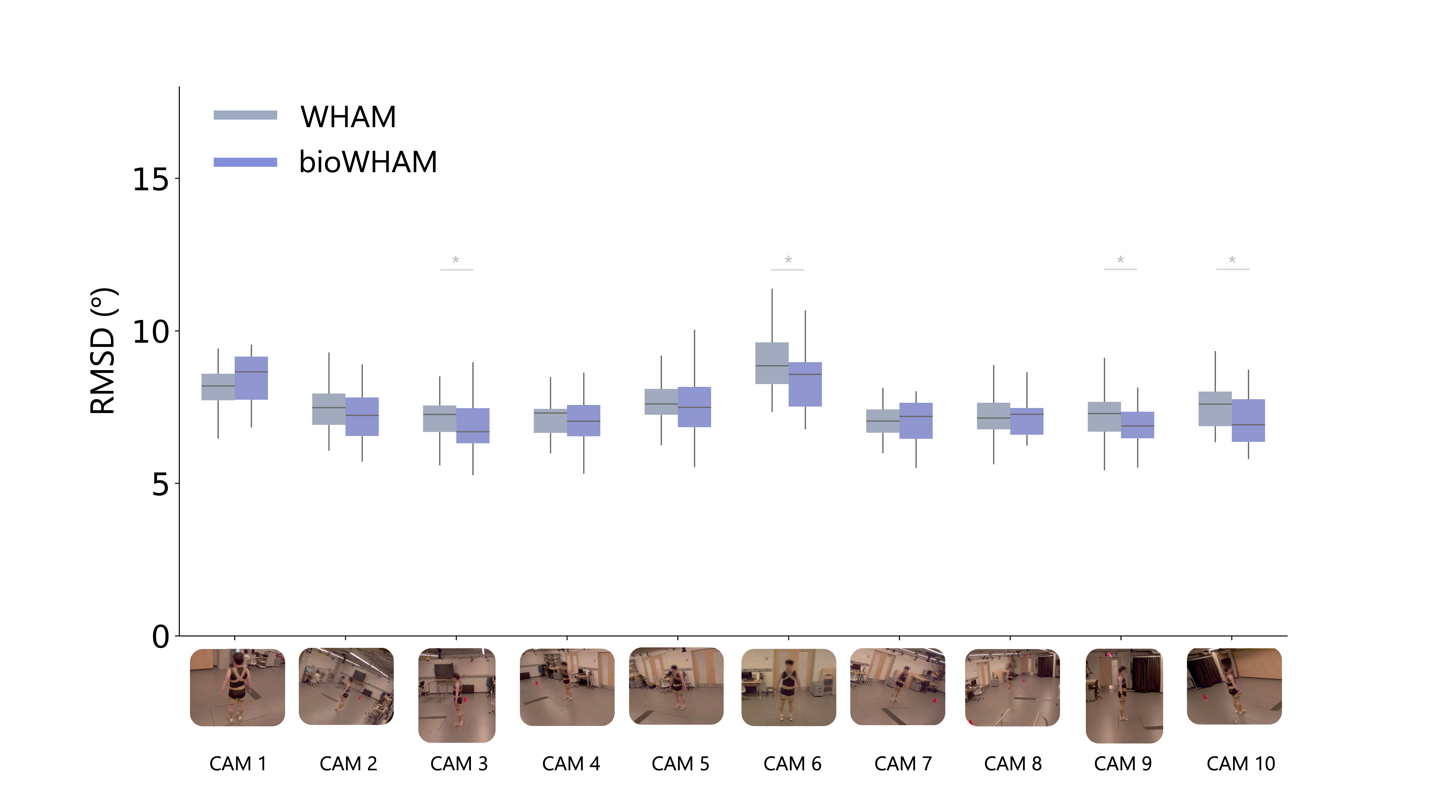


Fig. S7.  Effect of Biomechanical Modeling. Across the ten camera views, we found that the addition of biomechanical modeling (bioWHAM) did not consistently improve kinematics tracking accuracy. When improvements were observed, they were marginal (0.3$^{\circ}$ – 0.6$^{\circ}$). Overall, the differences for some cameras (e.g., Camera 3, 6, 9, and 10) were statistically significant (p < 0.005), even if not practically meaningful.
